# Use of the Internet and Mobile Phones for Self-Management of Severe Mental Health Problems: Qualitative Study of Staff Views

**DOI:** 10.2196/mental.8311

**Published:** 2017-11-01

**Authors:** Natalie Berry, Sandra Bucci, Fiona Lobban

**Affiliations:** ^1^ Division of Psychology and Mental Health School of Health Sciences, Faculty of Biology, Medicine and Health University of Manchester Manchester United Kingdom; ^2^ Health eResearch Centre Institute of Population Health University of Manchester Manchester United Kingdom; ^3^ Manchester Academic Health Science Centre Greater Manchester Mental Health NHS Foundation Trust Manchester United Kingdom; ^4^ Spectrum Centre for Mental Health Research School of Health and Medicine Lancaster University Lancaster United Kingdom

**Keywords:** psychosis, bipolar disorder, mHealth, eHealth, clinicians, mobile phone, Internet, intervention

## Abstract

**Background:**

Researchers are currently investigating the feasibility, acceptability, and efficacy of digital health interventions for people who experience severe mental health problems such as psychosis and bipolar disorder. Although the acceptability of digital health interventions for severe mental health problems appears to be relatively high and some people report successfully using the Internet and mobile phones to manage their mental health, the attitudes of mental health care staff toward such approaches have yet to be considered.

**Objective:**

The aim of this study was to explore mental health care staff experiences of clients with severe mental health problems engaging with the Internet and mobile phones to self-manage their mental health and their views toward these behaviors. The study also sought to examine the opinions expressed by mental health care staff toward digital health interventions for severe mental health problems to identify potential facilitators and barriers to implementation.

**Methods:**

Four focus groups were conducted with 20 staff working in mental health care services in the North West of the England using a topic guide. Focus groups involved 12 staff working in secondary care psychological services (7 participants in focus group 1 and 5 participants in focus group 4), 4 staff working in a rehabilitation unit (focus group 2), and 4 staff working in a community mental health team (focus group 3). Focus groups were transcribed verbatim, and transcripts were analyzed thematically to identify key themes that emerged from the data.

**Results:**

Four overarching themes, two with associated subthemes, were identified: (1) staff have conflicting views about the pros and cons of using Web-based resources and digital health interventions to manage mental health; (2) digital health interventions could increase access to mental health support options for severe mental health problems but may perpetuate the digital divide; (3) digital health interventions’ impact on staff roles and responsibilities; and (4) digital health interventions should be used to enhance, not replace, face-to-face support.

**Conclusions:**

This study is the first, to our knowledge, to qualitatively explore the experiences and attitudes of mental health care staff toward individuals with severe mental health problems using the Internet, mobile phones, and digital health interventions to self-manage their mental health. Understanding the positive and negative experiences and views shared by staff toward both current and potential digital health intervention use has enabled the identification of several considerations for implementation. Additionally, the findings suggest mental health care staff need clear guidance and training in relation to their responsibilities in recommending reputable and secure websites, forums, and digital health interventions and in how to manage professional boundaries on the Internet. Overall, the study highlights that digital health interventions could be well received by staff working in mental health services but importantly, such management options must be presented to frontline staff as an avenue to enhance care and extend choice, rather than as a method to reduce costs.

## Introduction

Mobile phone and Web-based psychological interventions, or digital health interventions, are increasingly being developed for people who experience mental health problems. Indeed, the National Institute for Health and Care Excellence has recommended the provision of computerized cognitive behavioral therapy (cCBT) for the treatment of depression and anxiety [1]. However, the Five Year Forward View, an initiative by the UK National Health Service (NHS) aiming to reform current services and transform care, highlights the current limited use of technology within services and sets out the priority to harness technology within clinical settings [2]. To this end, the NHS has approved a number of websites and mobile phone apps for a range of mental health problems [3] and has recently launched an early version of the NHS Digital Apps Library that catalogues a number of apps aimed to help people manage their health care needs [4].

Some individuals who experience severe mental health problems such as psychosis and bipolar disorder report already using the Internet and mobile phones to self-manage their mental health. For example, some use the Internet to search for health-related information such as medication, diagnoses, and symptoms and to discuss their mental health on the Internet with others [5-10]. Additionally, staff working in mental health care services have reported largely neutral or positive attitudes toward the use of digital health interventions for the management of mental health problems [11-17] but are cautious about using digital health interventions for severe and complex cases [15-17]. Specifically, many believe that digital health interventions could improve access to psychological interventions, increase comfort in disclosing information, normalize experiences and reduce stigma, monitor clients’ symptoms, evaluate therapeutic outcomes, and promote help-seeking behaviors [14-17]. However, staff also have numerous concerns about digital health interventions, including the perceived inferiority in comparison to face-to-face support, limited guidance with regard to efficacy and credibility, ethical concerns if clients report that they themselves or other people are at risk, potential breaches of data confidentiality, and limits in clients’ technology access and skills [13,15-17].

An evidence base is emerging regarding current Internet and mobile phone use for self-management reported by people with severe mental health problems, but there is a paucity of research examining the experiences and views of staff toward these behaviors. Additionally, although several studies have explored service user views regarding the hypothetical acceptability of digital health interventions for severe mental health problems (ie, the acceptability before or without receiving an intervention) [18], much of the current research investigating staff views is based on cCBT for mild-to-moderate mental health problems, rather than digital health interventions more generally for severe mental health problems. Digital health interventions based on approaches such as CBT [19], psychoeducation [20-22], and mindfulness [23] are being offered in a research context for people with severe mental health problems. However, to successfully implement digital health interventions, it is important to understand the views and concerns of staff who will be required to promote and support their use [24,25]. Therefore, this study aimed to (1) investigate the experiences and views of mental health care staff toward clients with severe mental health problems using the Internet and mobile phones to manage their mental health and (2) explore opinions expressed by mental health care staff (hypothetical acceptability) toward digital health interventions for severe mental health problems to identify the potential facilitators and barriers to the implementation of digital health interventions in mental health care services.

## Methods

### Design

Focus groups were used as a convenient way to explore a range of staff views while minimizing the burden of participation. Through the process of group discussion, they also facilitated the development and elaboration of ideas that may not have been previously articulated [26].

### Sampling and Recruitment

Participants comprised mental health care staff working in the NHS based in the North West of England and recruited via convenience sampling. Service leads working in mental health services were approached via email by the researchers enquiring as to whether they would be open to presenting their staff with the opportunity of participating in a focus group. The researcher then liaised with the service leads to arrange the focus groups with staff who had expressed an interest in participating. The lead researcher had no established relationship with any of the participants before the start of the study.

### Procedure

Four focus groups were held across three mental health trusts in the North West of England from April 2016 to September 2016. Focus groups involved staff working in psychological services, a community mental health team, and a rehabilitation unit and lasted between 30 and 60 min. Before the audio-recording of the focus groups, participants were presented with consent forms and a brief demographics and technology ownership questionnaire to complete. NB conducted all focus groups using a topic guide (Multimedia Appendix 1) in a private room in participants’ workplaces. Questioning focused on two key areas: (1) staffs’ experiences of clients’ use of the Internet and mobile phones and (2) views about the acceptability of implementing digital health interventions for severe mental health problems in mental health care services. NB kept field notes throughout focus groups, completed a reflective journal, and reviewed the topic guide and transcripts after each focus group to identify any additional areas of discussion that naturally arose.

### Data Analysis

Data were analyzed thematically to understand common themes arising in response to the research questions [27]. After the focus groups were completed and transcribed, NB (PhD student, psychology) read each transcript repeatedly for data familiarization and initially coded the transcripts in a cyclical process, returning to previous transcripts when new codes emerged. The other members of the research team (academic clinical psychologists SB and FL) also independently read and assigned codes to the first group transcript, and the team met to discuss and compare codes and develop an initial coding scheme. NB continued to develop this coding framework by analyzing the remaining transcripts and started to draw out preliminary subthemes emerging from these codes. Further team discussion was used to refine and create a final set of themes that reflected participants’ views and experiences across all focus groups. These themes were presented to some of the group participants, which helped to refine the way in which the themes were presented.

### Reflexivity

NB is a PhD student investigating how digital health interventions could be used to support people with severe mental health problems. SB and FL are academic clinical psychologists who are principal investigators on clinical trials implementing digital health interventions for this population and have extensive experience in conducting and supervising qualitative studies. It is important to acknowledge that these experiences may affect the analysis and interpretation of the data, so several steps were taken to minimize the likelihood of this occurring. First, NB was careful to present the research questions in an open and neutral way with no indication of the views of the research team and encouraging people to explore the full range of views. Additionally, questions surrounding the potential benefits and drawbacks of digital health interventions were initially phrased broadly to ask staff about their *thoughts* surrounding digital health interventions, and the terms *benefits* and *drawbacks* were only used later for further probing. Finally, NB kept a reflective journal to consider how staff responses in each focus group affected her own views about digital health interventions throughout data collection, analysis, and reporting and tried to take this into consideration when analyzing the data.

### Ethical Considerations

The study received ethical approval from NHS Cambridge South Research Ethics Committee (ref: 16/EE/0059). All participants provided verbal and written consent for participation, audio-recording of focus groups, and the use of direct quotations in publications resulting from the research. Participants did not receive any financial or professional incentives for participation.

## Results

### Participant Characteristics

A total of 20 mental health care professionals were recruited across four focus groups. A summary of participant characteristics is presented in Table 1.

### Thematic Analysis

**Figure 1:**
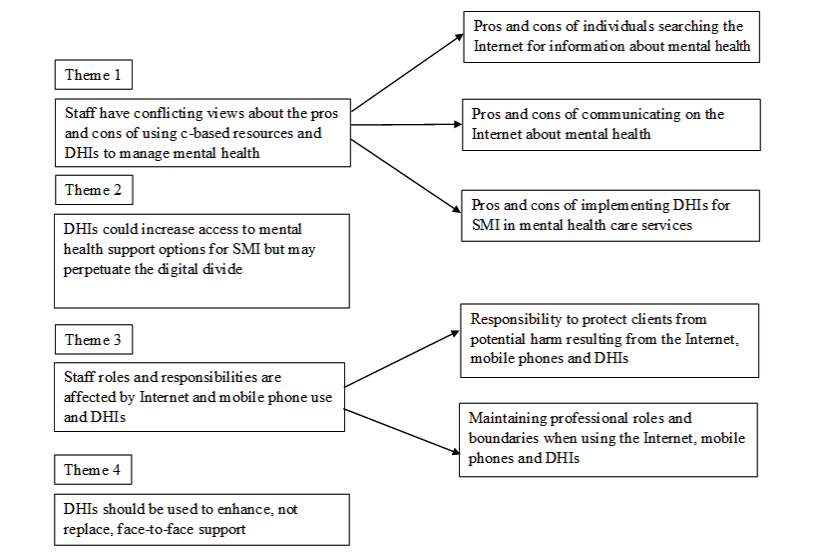
Diagram of themes.

Thematic analysis of focus group data generated four key themes and five subthemes: (1) staff have conflicting views about the pros and cons of using Web-based resources and digital health interventions to manage mental health; (2) digital health interventions could increase access to mental health support options for severe mental health problems but may perpetuate the digital divide; (3) digital health interventions impact on staff roles and responsibilities; and (4) digital health interventions should be used to enhance, not replace, face-to-face support. A diagram of the themes and associated subthemes is presented in Figure 1.

#### Theme 1: Staff Have Conflicting Views About the Pros and Cons of Using Web-Based Resources and Digital Health Interventions to Manage Mental Health

##### Subtheme 1: Pros and Cons of Individuals Searching the Internet for Information About Mental Health

Across all focus groups, staff welcomed clients searching the Internet for information about mental health problems because it allowed people to access potentially helpful information at any time and in any place, without the need to ask staff:

It’s instant for them at a time when they’re needing answers...It’s there at their fingertips. They don’t have to wait...until the clinic opens to speak to the CPN.

Participant 15, Focus group 3, Community mental health team

Indeed, several participants in both focus groups in secondary care psychological services described instances where clients had gone to extensive lengths to educate themselves about their mental health using information that had been retrieved on the Internet. This had sometimes been beneficial before beginning therapy:

...she’d done a lot of research herself...so when she came into therapy she was in a very different place than a lot of people because she kind of already started herself.

Participant 20, Focus group 4, Secondary care psychological services

**Table 1:** Summary of participant characteristics (N=20).

Demographic information				n (% or range)
**Overall sample**				
Mean age in years				42.35 (27-62)
	**Gender**			
		Female		16 (80%)
		Male		4 (20%)
	**Ethnicity**			
		White British		16 (80%)
		White Irish		2 (10%)
		British Pakistani		1 (5%)
		White other		1 (5%)
**Job role and technology ownership information**				
	**Focus group 1 (secondary care psychological services)**			
		**Job title**		
			Clinical psychologist	7 (100%)
		Mean time working in mental health services, years		14.67 (12-21)
		Mean technology comfort level		5.5 (4-6)^a^
		Mobile phone ownership		7 (100%)
		Smartphone ownership		7 (100%)
		Tablet computer ownership		4 (57%)
		Social media use		6 (86%)
	**Focus group 2 (rehabilitation unit)**			
		**Job title**		
			Staff nurse	3 (75%)
			Support worker	1 (25%)
		Mean time working in mental health services, years		14.5 (5.5-19)
		Mean technology comfort level		4.25 (1-6)^a^
		Mobile phone ownership		4 (100%)
		Smartphone ownership		3 (75%)
		Tablet computer ownership		3 (75%)
		Social media use		3 (75%)
	**Focus group 3 (community mental health team)**			
		**Job title**		
			Occupational therapist	2 (50%)
			Clinical practice nurse	1 (25%)
			Community team lead	1 (25%)
		Mean time working in mental health services (range)		12.75 (5-16)
		Mean technology comfort level		5.75 (5-6)^a^
		Mobile phone ownership		4 (100%)
		Smartphone ownership		4 (100%)
		Tablet computer ownership		4 (100%)
		Social media use		4 (100%)
	**Focus group 4 (secondary care psychological services)**			
		**Job title**		
			Clinical psychologist	3 (75%)
			Psychological therapist	2 (25%)
		Mean time working in mental health services		19.6 (15-25.92)
		Mean technology comfort level		5 (5-5)^a^
		Mobile phone ownership		5 (100%)
		Smartphone ownership		5 (100%)
		Tablet ownership		4 (80%)
		Social media use		3 (60%)

^a^1=extremely uncomfortable and 6=extremely comfortable.

Additionally, several clinical psychologists and psychotherapists in focus groups 1 and 4 had used Web-based resources within sessions with clients, which had been a valuable component of the session and helped normalize experiences:

There are some good YouTube videos...around compassion focussed therapy...we’ve done that together...so I guess if it’s done in a clinical setting in a careful way it’s been massively useful...in terms of normalising particularly and de-shaming.

Participant 6, Focus group 1, Secondary care psychological services

Across all focus groups, staff also revealed that they searched the Internet for information about their own mental and physical health care needs. Consequently, they understood and related to clients’ information-seeking and were generally willing to embrace, encourage, and, if needed, guide this behavior. Indeed, staff expressed that they would specifically want to see psychoeducation included within future digital health interventions. These comments reflect current Internet information-seeking behaviors by both staff and clients alike and suggest that staff would be positive about the provision of psychoeducational material in digital health interventions.

Although many of the experiences that staff shared were positive, concerns were raised about the abundance of unregulated Web-based material relating to mental health. Specifically, Web-based information could be biased, inaccurate, and misleading and, in all focus groups, staff described situations where clients had engaged in what they perceived as harmful or damaging behaviors as a direct result of reading information on unregulated websites:

I had a client in the past who bought... something...from America. He thought it was gonna cure his mental health problem...he ended up on the ward...but that was his belief that he read it on the Internet that if he got this substance it would make him better.

Participant 17, Focus group 4, Secondary care psychological services

Concerns were also expressed about Web-based information surrounding topics such as religion, conspiracy theories, and antipsychiatry messages. These were perceived as having the potential to reduce engagement with services and medication adherence and fuel distressing beliefs often associated with severe mental health problems:

...you can find information saying all psychiatry is the work of Satan and you can find sites that say don’t see a psychiatrist...

Participant 9, Focus group 2, Rehabilitation unit

It can feed your delusions.

Participant 11, Focus group 2, Rehabilitation unit

The term *trusted websites* was, therefore, mentioned frequently, with specific examples of charitable and national organizations. Private company websites such as pharmaceutical companies, private counseling and psychotherapy services, and unmoderated chat rooms were viewed as untrustworthy sources of information. Although staff noted that they themselves actively engaged in Web-based information-seeking, they had concerns about the vulnerability of clients. Specifically, staff felt that they had the training and experience to filter potentially biased or inaccurate information but that the general public (including clients) may not have such capabilities. Therefore, staff were keen to see the promotion of greater awareness regarding the potential dangers of unregulated websites.

In summary, staff expressed many positive and negative experiences of clients searching the Internet for information about mental health. Specifically, Web-based information had been a helpful resource before, and in-conjunction with, face-to-face therapy and could be accessed at any time clients needed information. However, negative experiences of clients accessing unhelpful Web-based content had led to concerns regarding the quality and trustworthiness of Web-based information.

##### Subtheme 2: Pros and Cons of Communicating on the Internet About Mental Health

Participants detailed many benefits of interacting with others via the Internet. In particular, staff in all groups had experienced clients’ receiving helpful support from peers via Web-based forums and social media websites. The anonymous nature of forums and the ability to communicate with others who had faced similar experiences were viewed as potential reasons why people may feel more comfortable expressing themselves via these platforms in comparison to face-to-face environments:

A lot of the forums you have pseudonyms and stuff don’t you...so you do feel more open and able to express yourself and your opinions more freely.

Participant 6, Focus group 1, Secondary care psychological services

Social media websites and forums were also viewed as a place where individuals could vent and feel like they had spoken with another person, even if they do not have a close social network, are unable to leave the house, or not comfortable speaking with others face-to-face:

People who struggle to relate to other people...they don’t have to leave their comfort zone in a way to almost be with people. It’s kind of an interim position perhaps.

Participant 18, Focus group 4, Secondary care psychological services

The positive experiences of social media websites and forums reflect the perceived utility of these resources as pathways to receive peer support and to connect with others with a shared understanding.

Staff in all focus groups were, however, concerned that discussing mental health problems on forums and social media websites could lead to individuals being bullied, trolled, or taken advantage of by others. One participant working in secondary care psychological services speculated whether the occurrence of negative Web-based behaviors such as cyberbullying could be because of the faceless nature of forums and some social media websites, which could lead to people not seeing the distress that negative comments can cause. Other participants acknowledged this viewpoint and agreed that clients they had seen had made similar observations:

Is there something about the internet though that can attract some negativity that people wouldn’t say in real life to people...you can sort of not see the impact of what you say...

Participant 5, Focus group 1, Secondary care psychological services

Staff across groups also described instances in which they felt clients had disclosed what they believed had been too much personal information on the Internet. They felt that such disclosures had the potential to cause embarrassment, distress, and lead to others targeting and taking advantage of the person who had posted this information. Of particular concern to all participants was that the Internet could increase opportunities to communicate with others in ways that were seen to enhance risk of self-harm and suicide:

We...had a bunch of girls who were communicating via Facebook...they all had a suicide pact together...

Participant 13, Focus group 3, Community mental health team

Staff suggested strategies that could be used to manage this, such as ensuring that forums are moderated. Staff working in a community mental health team even suggested that NHS Trusts should offer their own moderated forums for clients to engage with, reflecting the level of importance staff placed on forum provision and moderation.

In summary, staff believed the opportunity to connect with others with a shared understanding and the potential for accessible peer support was a benefit of Web-based communication and could be incorporated into future digital health interventions. However, staff had experienced or were concerned about the potential drawbacks of cyberbullying and trolling, overdisclosures, and Web-based communication about engaging in suicidal behaviors.

##### Subtheme 3: Pros and Cons of Implementing Digital Health Interventions for Severe Mental Health Problems in Mental Health Care Services

The anonymous nature of digital health interventions was viewed as beneficial in comparison to face-to-face support because there may be times and situations where individuals feel more comfortable disclosing sensitive information to a digital device, rather than another person. Additionally, staff in the community mental health team and secondary care mental health services observed that clients could sometimes be reluctant to complete paper-based exercises because of concerns about others finding these materials. Therefore, people may feel more comfortable using digital health interventions because of the increased privacy and reduced risk of others being able to find hard copies of therapy materials:

We’ve had clients who have wanted to record but are fearful of somebody finding paper so I think it could give some sort of privacy confidentiality...

Participant 20, Focus group 4, Secondary care psychological services

However, staff in all focus groups raised fears that companies or individuals could be able to hack digital devices and obtain sensitive user information and responses. These concerns reflect the dilemma that although digital health interventions may increase privacy, this may still be limited because of the potential security issues associated with using technology.

Staff were also asked about the use of digital devices to monitor thoughts, feelings, and experiences in-the-moment. Responses to symptom monitoring via apps were largely positive and staff across groups identified times when clients had found it difficult to remember how they had been feeling since the last session or appointment they attended. Therefore, digital devices were viewed as a potential method for people to record symptoms and experiences in the moment, offering the opportunity to discuss and reflect on over time. Indeed, one staff member specifically searched for an app for a client to help identify triggers and patterns:

...I was sort of looking at that as a way of getting her to monitor her moods over a period to try to understand a bit better the pattern of what was happening with her and why.

Participant 14, Focus group 3, Community mental health team

However, staff working in secondary care psychological services and a community mental health team felt continuous monitoring may become tiresome and could lead people to unhelpfully dwell on experiences. Therefore, the suggestion was made that monitoring via digital devices should also involve recording positive events for people to identify, recognize, and acknowledge.

Across all focus groups, digital health interventions were seen as useful for some people because it may be easier for them to be honest about their feelings when asked on a faceless device rather than by another person. Conversely, concerns were raised that the faceless nature of digital health interventions may lead to people underreporting the severity of their symptoms to reduce the levels of care they receive, although others might exaggerate symptoms to increase care:

...you don’t know whether that person’s racking it up...

Participant 9, Rehabilitation unit

...it could be like I have no symptoms no problems right now, can I get out of hospital now?

Participant 11, Rehabilitation unit

Staff responses regarding the potential benefits and concerns about digital health interventions reflect the mixed views toward this approach. Specifically, concerns regarding data confidentiality and truthfulness of responses need to be addressed to ensure that staff feel comfortable recommending clients to receive digital health interventions.

#### Theme 2: Digital Health Interventions Could Increase Access to Mental Health Support Options for Severe Mental Health Problems but May Perpetuate the Digital Divide

Regular Internet and mobile phone use was viewed as the norm, particularly for the younger generation and, therefore, seen as a mechanism to improve access to mental health support. However, there was the recognition that many people do not have the technology skills required to use digital health interventions. Staff working in a residential unit noted that many clients showed very limited technology skills and feared this would pose a significant barrier to providing digital health interventions within services, thus perpetuating the digital divide:

I do get asked quite a lot. I’ve got a phone I don’t know how to send a text, can you send a text for me? But it’s that basic...

Participant 8, Focus group 2, Rehabilitation unit

A technology skills training program was suggested as one possible solution to overcoming this barrier. Staff also revealed that some clients did not have ownership of, or access to, the Internet or mobile phones and even those who did would lose their phones and change numbers frequently. These perceptions led to concerns about how individuals would be able to access digital health interventions. When asked about the NHS supplying devices for digital health interventions, staff in all groups felt the NHS should not provide the required technology because of concerns that tablets and mobile phones may get lost, sold, or damaged. Additionally, staff in a rehabilitation unit and community mental health team believed that other health care needs such as medication should take precedence over the provision of digital health interventions. Conversely, a smaller number of participants within these groups argued that digital health interventions could allow more people to have access to support options, which could reduce the levels of staff needed and save the NHS money. The reflective journal completed by the interviewer detailed the observation that the NHS funding digital health interventions was a particularly contentious issue and raised animated responses from all participants. A note made in the journal after the focus group with the community mental health team reflects this point:

I am beginning to see a pattern emerging with participants displaying particularly strong views towards the idea of the NHS funding devices. As soon as the digital divide barrier is raised by participants, the view that the NHS should not pay for devices is raised almost immediately. When I then try to explore why, this question is met with polite laughter at the thought that the NHS should make these provisions.

Interviewer, Reflective journal, Focus group 3, Community mental health team

Therefore, the overall position of staff was that the NHS would not have the resources required to supply the devices needed to deliver digital health interventions.

Although potential issues regarding access and capacity were raised, participants in all focus groups were still able to recall experiences of clients actively engaging with digital devices to self-manage their mental health, including (1) accessing information about medication, diagnoses, symptoms, personal stories, and coping strategies; (2) using forums and social media websites to discuss mental health; (3) using mobile phone cameras to photograph formulations during therapy sessions; (4) using alarms and calendars on mobile phones for appointment and medication reminders; and (5) using apps to receive already existing self-management options.

Findings from this theme suggest the digital divide may be a barrier that prevents mental health care staff from supporting the implementation of digital health interventions into clinical practice. Specifically, concerns were raised by participants about clients’ basic technology skills and ownership, and staff questioned how some would be able to afford access to digital health interventions. However, this theme also identified the potential facilitator to implementation that some individuals and staff are already using digital devices for self-management.

#### Theme 3: Digital Health Interventions’ Impact on Staff Roles and Responsibilities

##### Subtheme 1: Responsibility to Protect Clients From Potential Harm

Staff reported a strong sense of responsibility toward clients engaging with digital devices. This was particularly evident in a focus group involving participants working in a residential unit that was soon to be serviced with a tablet computer for use by clients. Staff felt they would need to limit the amount of time that clients could access the tablet, monitor and control websites and apps visited, and conduct risk assessments before allowing access:

We’d supervise, we’d restrict, we’d filter, we’d feedback...

Participant 11, Focus group 2, Rehabilitation unit

This perceived responsibility seemed to stem from fearing that clients may use the tablet to access websites containing potentially inappropriate content such as extreme beliefs written by others, antipsychiatry messages, pornographic material, and gambling websites, which may exacerbate symptoms and decrease engagement with services. However, 2 participants noted that, despite these concerns, access should still be provided to ensure that clients are given the opportunity to live autonomous lives. Additionally, prior experiences of clients writing status updates on their social media profiles surrounding delusional beliefs had led to staff in this service needing to intervene by contacting social media websites and restricting client access. Therefore, it was apparent that staff felt they needed to protect some clients’ because of their perceived vulnerability to Web-based content and overdisclosure. The reflective journal completed by the interviewer after this focus group detailed the emotional response from participants:

I get the sense that staff feel a huge burden of responsibility towards protecting their clients from harm and take this responsibility incredibly seriously. Staff expressed strong concerns that they would be required to supervise clients using the tablet, when their time would be better spent elsewhere. It felt like staff believed tablets could be positive, but the limited staff resources would mean the provision of tablets at this time would be a burden for staff; not a helpful addition. Whilst conversations remained positive, two participants did raise their voices and spoke emotionally about their fears regarding the additional responsibilities and work pressures associated with the acquisition of a tablet for the unit.

Interviewer, Reflective journal, Focus group 2, Rehabilitation Unit

Staff participating in the focus groups within secondary care psychological services and a community mental health team also raised concerns about perceived responsibility. For example, several staff said they would recommend reputable websites or apps to clients; however, others were uncomfortable making recommendations because of their responsibility if these resources were unhelpful. Indeed, in one focus group, participants expressed a wish for more detailed information about NHS-endorsed websites and apps that they could recommend to clients:

If I was thinking of an app for a service user, you’re a bit uncomfortable recommending...

Participant 14, Focus group 3, Community mental health team

That’s what we were taking about developing weren’t we something like a directory of things that we could use...

Participant 15, Focus group 3, Community mental health team

Additionally, staff in all focus groups were worried about their moral, legal, and professional obligations with regard to assessing risk information such as suicidal ideation and behaviors if clients were monitoring symptoms via digital devices:

...if somebody’s really low and threatening suicide, what responsibility do you have for that; what would be their expectations?

Participant 19, Focus group 4, Secondary care psychological services

What if it gets to you when you’re in an appointment and you can’t respond to it until the next day?

Participant 18, Focus group 4, Secondary care psychological services

A potential solution identified by staff working in secondary care services and a community mental health team was that clients could bring their own symptom reports to appointments. Not only did staff feel this would give clients control over the information they shared, they also felt the level of burden and responsibility on themselves would be minimized. This proposal received particularly strong responses from all participants in these focus groups, as noted in a comment in the reflective journal made by the interviewer after the fourth focus group:

Staff made the really interesting suggestion in the first focus group that rather than receive symptom monitoring responses automatically, they would prefer to receive them from the client to address the power imbalance and minimise the burden associated with automatic responses. Therefore, I decided to explore this further in subsequent focus groups. Participants seemed particularly animated and excited at the potential for technology to be used in this way, which was demonstrated through non-verbal communication such as nodding in agreement and smiling and through verbal acknowledgements of agreement. These positive responses were a stark contrast to the proposal of automatic symptom monitoring, which generated immediate disapproval from all but one participant across groups

Interviewer, Reflective journal, Focus group 4, Secondary care psychological services

Staff working in secondary care services also described devoting time within sessions to reflect on friendships clients had formed on the Internet over topics such as suicide pacts and self-harm strategies. Additionally, staff in these focus groups had experiences of providing psychoeducation to address misinformation that clients had obtained on the Internet. These past experiences had contributed toward concerns about the availability of unregulated Web-based material and Web-based discussions surrounding mental health.

To summarize, staff recounted several experiences of clients accessing Web-based content that led to negative consequences. Therefore, staff felt paternalistic toward clients’ access to this content. Additionally, concerns regarding their own knowledge of websites and apps prevented some from making recommendations and concerns raised about the potential legal, moral, and ethical implications regarding automatic symptom monitoring need to be considered during implementation.

##### Subtheme 2: Maintaining Professional Roles and Boundaries

Staff were not directly asked about how the Internet and mobile phones affected their professional boundaries with clients; however, the issue naturally arose during all focus groups and was discussed at length. For example, participants reported concerns about clients sending friend requests over social media websites, the availability of personal information on the Internet that may affect professional relationships with clients, and fears that others may see personal social media posts that they disagree with and subsequently report. For this reason, many staff said they did not use social media websites or limited the amount of personal information they disclosed on the Internet.

Staff in focus group 1 (secondary care psychological services) also detailed situations in which they had used text messages (short message service, SMS) to remind clients about upcoming appointments; although, one participant noted this would only be for the first few appointments to avoid taking too much responsibility. Additionally, there were differing opinions expressed by community mental health team staff regarding sending text messages to clients. Some staff shared their personal mobile phone numbers, with the understanding that there would be limits as to when clients could contact them, whereas others were concerned about breaches in data confidentiality and the risk of clients contacting them outside working hours. Indeed, the focus group transcript and reflective journal maintained by the interviewer revealed a debate between participants regarding the boundary issues associated with staff and client mobile phone communication:

I text a few of mine...I don’t do it with everybody but they do respond well to it...

Participant 12, Focus group 3, Community mental health team

I think you have to be very careful what data is relayed in a text. If it’s simple facts of phone numbers or dates and times fine.

Participant 13, Focus group 3, Community mental health team

...and they have your number then so you don’t want to be giving it out to people who might be contacting you.

Participant 11, Focus group 3, Community mental health team

Monitoring clients’ social media profiles for information about daily functioning and risk was unanimously criticized in all groups as a misuse of trust and power, which could potentially damage the therapeutic relationship.

I think it could actually be quite damaging...especially if your clients have quite paranoid thoughts..

Participant 3, Focus group 1, Secondary care psychological services

You should respect that everything they want to bring to that session that’s what they want to talk about...

Participant 2, Focus group 1, Secondary care psychological services

The suggestion of staff accessing clients’ social media profiles was the topic that seemed to provoke the most emotive responses from participants during focus groups. The interviewer noted in the reflective journals that responses often involved verbal utterances of disagreement such as tutting and shocked laughter and nonverbal cues such as shaking heads and raising eyebrows. The importance participants placed on this topic was also reflected in focus group 1 responses to the interviewer asking how they found taking part in the research:

Fine particularly that social media [question]...

Participant 6, Focus group 1, Secondary care psychological services

Especially when it comes to researchers who are obviously very very far removed from the real world or (.) working with people with complex mental health problems on therapy you know they might think things are a good idea...so if there’s any way that we can kind of you know just inform their thinking a little bit.

Participant 7, Focus group 1, Secondary care psychological services

Additionally, staff wanted to receive mobile phone symptom assessments directly from clients themselves rather than automatically via an app, and only one participant outwardly expressed the preference for receiving these automatically. This was so clients should take ownership of their data and choose what to share, thus ensuring that staff were not placed in the expert role.

#### Theme 4: Digital Health Interventions Should Be Used to Enhance, Not Replace, Face-to-Face Support

The self-directed nature of digital health interventions was also viewed by all groups as an empowering way people could take control of, and responsibility for, their own mental health care needs:

It gives them some control doesn’t it. It can be empowering...

Participant 16, Focus group 4, Secondary care psychological services

However, across groups, staff held the unanimous belief that digital health interventions should never be offered as a replacement to face-to-face support. Instead, it was suggested that digital health interventions should be used to extend support options available. For example, one participant suggested that app-based symptom monitoring could be implemented by services as a method for routine outcome monitoring to evaluate changes throughout therapy. Additionally, staff working in secondary care psychological services suggested that digital health interventions could be used at the end of therapy to allow clients to access coping mechanisms and strategies they had developed during sessions:

The ones that do...the best in follow up are the ones that have kept their letters and maps and have kept everything kind of accessible and have continued to use them so I guess that these could be transferable to some sort of technology.

Participant 19, Focus group 4, Secondary care psychological services

Staff in both focus groups in secondary care services were against the idea of clients using solely self-directed digital health interventions because of fears that individuals would be left alone to deal with any issues that surface. Therefore, they suggested that members of their care team might be able to support clients using digital health interventions during routine home visits. Interestingly, staff working in a community mental health team were willing to support engagement with digital health interventions; one participant suggested that staff could take tablet computers to clients’ homes to work through digital health interventions together:

...if there were tablets that could work outside the community that we could show and go through the process to show how simple it (an app) is and what they could potentially gain from it, I think there could be a definite place for it.

Participant 15, Focus group 3, Community mental health team

The novel ideas described by participants reflect their overall view that digital health interventions have the potential to be used within existing services, but there was a strong view that digital health interventions should not replace in-person support:

It shouldn’t be used to replace face to face, but it should be used to enhance.

Participant 8, Focus group 2, Rehabilitation unit

Such views seemed to stem from the need for a strong therapeutic relationship in delivering support for people with severe mental health problems, and staff expressed the concern that digital health interventions could not and should not attempt to mimic this relationship:

I’ve seen computer programmes where it almost tries to offer a therapeutic relationship and it gives kind of fake empathy...It is terrible and it kind of made me a bit annoyed just watching it...

Participant 5, Focus group 1, Secondary care psychological services

Staff in secondary care psychological services also noted that you can never take a *one size fits all* approach in therapy and that clinical formulations are needed. Therefore, staff were concerned that digital health interventions would not allow the personalization needed to deliver effective therapy. Cautions about digital health interventions were also based on previous experiences of cost-cutting strategies implemented in NHS services, and staff were therefore concerned that digital health interventions may be used as an excuse to reduce staffing costs and care provisions in severe mental health problems:

...it’s substituting proper therapy for something that isn’t proper therapy and anticipating or hoping that people will get better and it being a way of actually saving money and resources.

Participant 16, Focus group 4, Secondary care psychological services

The strong position held by staff regarding their views that digital health interventions should never attempt to replace face-to-face care was reflected in the repetitious comments made about this viewpoint. The interviewer noted in the reflective journal that the recurring nature of these comments indicated that this was a significant potential factor affecting the likelihood of implementation:

Following initial reviews of these transcripts, I am yet again struck by the repetitive viewpoint expressed by staff that we should never attempt to replace in-person support with DHIs. During focus groups, it felt like staff could list a significant number of benefits of DHIs, but this overarching concern led to caution. If staff hold such dogmatic views about this issue, it may be a significant barrier to the implementation of DHIs in secondary care services.

Interviewer, Reflective journal, Data analysis

## Discussion

### Principal Findings

This study sought to examine the experiences and views of mental health care staff toward clients with severe mental health problems using the Internet and mobile phones to manage their mental health and the hypothetical acceptability of digital health interventions for severe mental health problems to identify facilitators and barriers to implementation in secondary care services. Staff had a wide range of both positive and negative experiences of clients with severe mental health problems using the Internet and mobile phones for self-management, and staff were cautious, but optimistic, about the implementation of digital health interventions.

Web-based information-seeking was viewed positively and staff welcomed the incorporation of psychoeducation material into digital health interventions, suggesting that this could be well received by staff and service users alike. Concerns surrounding the abundance of unregulated Web-based material echo previous qualitative work, where individuals experiencing severe mental health problems also queried the trustworthiness of information from Internet sources and preferred information from organizational and charitable websites, rather than private companies or chat rooms [5,9]. Such skepticism surrounding the reliability of information found on the Internet may be warranted. A recent search for schizophrenia-related videos on the video-sharing website YouTube, revealed that only 34% accurately portrayed schizophrenia [28]. Additionally, mental health-related information on the Internet is reportedly of poor quality [29], and many websites are biased toward providing information about biological causes and medical treatment options [30]. However, a more recent study has reported that the quality of Web-based information specifically for severe mental health problems is of relatively good quality [31]. It has been suggested that health care professionals should direct clients to appropriate trusted websites to combat issues regarding the reliability of Web-based information [32]. In this study, staff endorsed this suggestion; however, some were concerned about their own limited knowledge surrounding websites and apps. Therefore, some participants expressed a need for a catalogue of evidence-based and NHS-endorsed resources they could recommend to clients.

Recently, the NHS introduced an early version of the once defunct NHS Digital Apps Library that contains a list of NHS-approved apps for numerous physical and mental health care needs [4]. Globally, the American Psychiatric Association has developed an evaluation model for use by staff to determine the appropriateness of apps for clients [33], and in Australia, the mindhealthconnect website, supported by the Australian government, lists a range of *trusted* Web-based tools and apps for people to self-manage their mental health and well-being [34]. Given the enthusiasm of participants to receive information regarding credible websites and apps, it is likely that these resources would be helpful for staff to review and implement. Therefore, efforts must be made to ensure that staff are made aware of, and encouraged to use, these new resources.

It has been suggested that social media websites and forums could be used to deliver interventions and provide peer support options for people experiencing severe mental health problems [35,36]. Early findings have indicated that this approach could be feasible and acceptable [37-39] and that individuals already actively access these platforms to receive support [40,41]. Although staff outlined several fears about clients engaging with social media websites and forums, they also described situations where clients had been able to connect with others on the Internet with a shared understanding. Therefore, the use of social media websites and forums to deliver interventions may be valued and utilized by people with severe mental health problems, although staff concerns such as forum moderation must be considered for successful implementation. Additionally, researchers have proposed that social media profiles may contain valuable information about individuals’ daily lives and functioning, which could be a useful tool for clinicians to make assessments and diagnoses [42]. Staff were unanimously against viewing clients’ social media profiles and viewed this behavior as a misuse of power; therefore, staff attitudes would be a significant barrier to the implementation of this approach in services.

Staff appeared to be paternalistic toward clients’ access and use of the Internet and mobile phones, with a perceived need to guide clients toward the right information; this was particularly true for staff working in a rehabilitation unit. Gatekeeping and paternalism by staff is not restricted to Internet access and digital health interventions. For example, staff have also been found to be paternalistic when deciding whether to refer clients to clinical trials [43] and treatment options clients should receive [44]. Involving clients in a shared decision-making process with regards to Internet, mobile phone, and digital health intervention access is therefore key, and rather than preventing access, clients should instead be encouraged to speak with staff to help make decisions regarding access together.

Although some staff believed that digital devices could increase access to evidence-based interventions, concerns regarding client access and ability to use such devices were raised. These perceptions about technology access and ownership somewhat contradict recent findings in the field. For example, a recent meta-analysis indicated a narrowing gap in mobile phone ownership between the general population and individuals experiencing psychosis [45]. Numerous studies since this review have indicated relatively high technology access and ownership by people who experience severe mental health problems [46-48]. However, although there has been a reduction in the digital divide since 2011, some people with severe mental health problems remain digitally excluded [49]. Therefore, those delivering digital health interventions should remain mindful of access issues within this population and ensure digital exclusion is minimized. For example, staff suggested that technology skills training programs could be offered to ensure people are able to fully engage with digital health interventions. Additionally, staff did not feel the NHS should provide digital devices for people to receive interventions because of fears that clients may lose or sell mobile phones and tablets. In contrast to this viewpoint, technology return rates have been high (86% and 95%, respectively) in two studies where digital devices were provided for participants with severe mental health problems [50,51]. This suggests that staff concerns regarding individuals’ capabilities of maintaining and returning digital devices may be inaccurate, and researchers should report device return rates to determine whether such concerns are warranted.

Staff were generally optimistic toward using mobile phone apps for symptom monitoring but expressed concerns about their responsibility when receiving symptom reports from clients’ because of the fear of missing risk disclosures. Therefore, staff stated a preference for receiving symptom reports from clients directly within sessions, rather than automatically. This somewhat contradicts the current direction of mobile phone apps for symptom monitoring in this population, which, although can be used by clients to share with their care team, tend to deliver symptom reports to a central server for staff to use to identify indications of relapse [52,53]. Therefore, issues surrounding the legal and moral responsibilities of staff when viewing automatic symptom reports and their comfort in implementing such approaches in practice need to be considered.

Staff in all groups repeatedly expressed the concern that digital health interventions could not, and should not, replace face-to-face care and should instead be used as an adjunct and as a method to extend choice. Such fears seemed to stem from the belief that the therapeutic relationship between client and therapist is key, and digital health interventions could never replace or mimic this relationship. There is some evidence to indicate that individuals can form a positive therapeutic alliance with self-directed digital health interventions [54,55]. However, further work is required to fully understand the therapeutic relationship in the context of digital health interventions to address the concerns expressed by staff.

### Study Limitations

Findings must be interpreted in the context of some limitations. First, half the sample were clinical psychologists. Therefore, experiences of clients engaging with websites and apps and views toward using digital health interventions may be different to individuals working in other roles. Participants were mental health care staff working in the NHS in the North West of England; implications regarding implementation are, therefore, limited to NHS mental health services. Participants reported generally high levels of comfort using technology themselves, which may have resulted in them finding digital health interventions more acceptable than staff who are less comfortable using technology. Staff involved in each focus group were part of the same team and, in all cases, the service lead also participated in the focus group. Therefore, staff may have been more cautious when sharing information about their views than if focus groups had involved staff with no prior relationships. Conversely, the close working relationships observed within each focus group may have enabled participants to feel more comfortable speaking openly and honestly about their views. To explore this potential limitation further, the reflective journals kept by the interviewer were reviewed for commentary about the group dynamics observed. Specifically, positive interactions during all focus groups and the absence of any conflicts between members were noted. Furthermore, the interviewer noted that service leads did not differ from other participants in the duration or number of experiences expressed and all the participants seemed to welcome and acknowledge opposing viewpoints. A strength of the focus group design is that it allows people to generate ideas through discussions with each other. However, an associated limitation with this approach is that the data generated are dependent on the individuals within each group, so individual perspectives may not be discussed and social pressures may impede members giving differing opinions from the group consensus. However, to minimize the interdependency of participants, group dynamics were managed by the interviewer so that each group member had the opportunity to present their views, and the interviewer kept a reflective journal throughout data collection and analysis to reflect on group dynamics. Finally, because of the practicality and time pressures for mental health care staff taking part in focus groups, we were advised to conduct focus groups within teams, rather than invite mental health staff to separate focus groups. Ideally, sample characteristics across focus groups are homogenous; however, to minimize participant burden, we accepted groups would be heterogeneous in nature. That said, across all four focus groups commonalities in experiences and viewpoints were stark despite differing job roles and any differences in viewpoints were noted in the results section

It is often considered best practice to return interview transcripts to participants for member checking. It was not possible to return transcripts to participants for this purpose because of the potential breaches in privacy and different viewpoints presented within focus groups. Finally, the research team have previous and current involvement in projects implementing digital health interventions for severe mental health problems. Although the researchers were mindful about reducing potential biases, it is important to acknowledge that such experiences may still affect the interpretation and analysis of data.

### Implications for Clinical Practice and Implementation

The concerns raised by staff regarding client access to potentially harmful Web-based content reflects the need for a comprehensive, accessible, and widely disseminated resource containing links to approved websites for clients to access. Additionally, some staff expressed limited knowledge about websites and mobile phone apps they could recommend to clients and were concerned about making unhelpful recommendations. Therefore, Web-based libraries containing information about approved websites and apps such as the NHS Digital Apps Library [4] must be presented to staff as a potential resource they can use to identify relevant options they can recommend to clients. Additionally, paternalistic viewpoints were expressed toward clients accessing the Internet and mobile phones and staff acknowledged that Web-based misinformation and communication had often needed to be addressed in therapy. Therefore, mental health care staff must be prepared to explore and address these issues in clinical practice. Furthermore, the exploratory nature of the qualitative methodology led to the identification of new and interesting staff perspectives that have not yet been explored. Therefore, researchers could use the viewpoints identified in this study to inform the design of future surveys to explore quantitatively whether these views are prevalent on a larger scale.

This study also reports numerous facilitators and barriers to the implementation of digital health interventions for severe mental health problems in secondary mental health care services. Staff views align with the general theory of implementation, which details the key constructs that influence the implementation of complex interventions in health care settings [56]. These findings in relation to this theory suggest that technology skills training for staff and clients alike must be delivered to foster capability. Additionally, further reporting of technology return rates in clinical trials delivering digital health interventions and considerations for funding devices in service settings are needed to ensure capacity for implementation. The cautious optimism shown by staff suggests there is the potential to implement digital health interventions for severe mental health problems in secondary care services, but the identified barriers must be considered and addressed before implementation. Digital health interventions for severe mental health problems are not routinely offered in treatment because of the need to establish a more concrete evidence base. Therefore, clients are often only referred to these management options as part of a clinical trial. Although the exploration of continuous investment by staff cannot currently be determined, if digital health interventions are to be implemented within secondary care services, examination of continuous contributions by staff must be made.

### Conclusions

This study is the first, to our knowledge, to qualitatively explore the experiences and attitudes of mental health care staff toward individuals with severe mental health problems using the Internet, mobile phones, and digital health interventions to manage their mental health. Findings showed that staff had both positive and negative experiences of using the Internet and mobile phones for self-management. Additionally, a range of facilitators and barriers to implementation were identified. Although staff were generally positive about current use and able to detail many experiences where clients had found engaging with these resources helpful, some concerns were expressed regarding trustworthy websites and the security of digital health interventions. Therefore, continued and improved identification and cataloguing of evidence-based resources on the Internet and digital health interventions must be made to facilitate staff comfort in referring clients to manage their health care needs digitally. Staff approached the idea of digital health interventions with cautious optimism, but concerns regarding legal and moral responsibilities and fears over a diminished therapeutic relationship must be addressed before implementation. Importantly, staff endorsed the provision of digital health interventions for severe mental health problems as an adjunct to face-to-face support but held the fear that digital health interventions would be used as a cost-cutting strategy. Therefore, to ensure implementation, digital health interventions should be presented to frontline staff as a tool to enhance care and extend choice.

## Appendix

Multimedia Appendix 1 Topic guide used in focus groups with mental healthcare staff.

## Abbreviations

cCBT: computerized cognitive behavioral therapy
NHS: National Health Service
