# Supplementary material for: Use of the Internet and Mobile Phones for Self-Management of Severe Mental Health Problems: Qualitative Study of Staff Views
Source: JMIR Ment Health. 2017 Nov 1;4(4):e52. doi: 10.2196/mental.8311 (PMC5688247; doi:10.2196/mental.8311)
Supplement: Multimedia Appendix 1 [file mental_v4i4e52_app1.pdf]

Interest and use of technology for self-management purposes by individuals experiencing severe mental health problems: service user and clinician perspectives.

Interview guide: clinicians.

Version 2: 04/08/16

Principal Investigator

Natalie Berry (PhD student)
Room S42, Zochonis Building
School of Psychological Sciences
Brunswick Street
University of Manchester
Manchester
M13 9PL
Tel: 0161 306 0428
Email: natalie.berry@manchester.ac.uk

Supervisory Team

Dr Sandra Bucci, School of Psychological Sciences, University of Manchester.
Professor Fiona Lobban, Spectrum Centre for Mental Health Research, Lancaster University.

**Equipment:**	Participant Information Sheet<br>Consent forms<br>Encrypted audio-recorder<br>Demographics questionnaire

**Prior to interview:**	Participant Information Sheet – any questions?<br>Consent form – explain audio recording and what is discussed in focus groups must remain confidential.<br>Demographic data (separate sheet)

**Introduction:**	Introduce self, welcome, and thank participants for attending the focus group. Ensure that the participants are comfortable.
**Consent:**	Re-confirm informed consent is still valid and participants still wish to take part.
**Focus group details:**	Outline focus group procedures and expected length of time for the focus group.<br>Remind the participant that the interview will be audio recorded, that participation is voluntary, that they have the right to withdraw at any point without penalty and offer pauses and breaks.
**Confidentiality:**	Explain confidentiality – everything the participants say during the focus group will remain confidential and that participants should not share what others have said during the focus group with others.
**Explain purpose of the study and take any questions:**	“Thanks again for meeting with me today. The focus group is split into two parts. In the first half we will discuss what sorts of things your clients might use the internet and mobile phones for and any experiences you have with clients using technology to support their mental health. In the second half we will move on to talk about your thoughts, views and ideas of people receiving mental health support online and through mobile phones. The focus group will take around 45 minutes to complete. With your permission, the focus group will be audio-recorded and then typed up to make sure we have an accurate summary of what you have said. Recordings will be securely stored in password protected computer files. Sometimes I may use quotes that you provide during the interview in publications, but I would always make sure that you would not be identifiable from these quotes. Do you have any questions at all?”
**Demographics questionnaire:**	Remind participant that the focus group is not being recorded yet and ask participants to complete the demographics questionnaire.

Section 1: Current technology use to support mental health

**Domain**	
**Information-seeking online:**	"What are your experiences of clients using the internet to find out information about mental health?"<br>***prompt****: what sort of information did they search for?*<br>***probe****: what did you think about the information that they found?*<br>***probe****: were there any websites that they found particularly helpful?*<br>***probe****: were there any websites that they found particularly unhelpful?*<br>***prompt****: what are the positive things about searching for information about mental health online?*<br>***prompt:*** *have clients ever had any negative experiences when searching for information about mental health online?*
**Talking about mental health online:**	"What are your experiences of clients talking about their mental health on the internet?"<br>***prompt:*** *social media (e.g. Facebook, Twitter), blogs, forums*<br>***probe:*** *what are your thoughts about people using the internet to talk about mental health problems?*<br>***prompt:*** *what might be the benefits for people speaking about mental health online?*<br>***prompt:*** *do you have any concerns about people speaking about mental health online?*
**Other ways people use the internet for their mental health:**	"Are there any other ways your clients have used the internet for their mental health?"

**Other ways people use mobile phones for their mental health:**	“What is your experience of clients using mobile phones to support their mental health?”<br>***prompt:*** *appointment reminders, medication reminders, smartphone applications*<br>***if yes -*** ***probe:*** *would you be able to tell me a bit more about that?*<br>***probe:*** *how was that helpful for the client?*<br>***probe:*** *did the client ever had any negative experiences when doing that?*<br>***if no -*** ***probe:*** *are there any reasons why you don’t think your clients use mobile phones to support their mental health (e.g. appointment reminders, medication reminders, smartphone applications)?*

Section 2: Concept of technology-delivered interventions for SMI

**Introduce concept:**	“So we’re now moving into the second part of the interview. In this part we will be speaking about your thoughts, views and ideas about support options that could be offered on mobile phones applications or on websites for people who experience severe mental health problems. These interventions often do not involve a clinician or support person, are entirely self-directed and may be used alone or in conjunction with face to face therapy. Recent examples are CBT-informed mobile apps and online programmes for psychosis and bipolar disorder, online psychoeducation and online and mobile-phone delivered mindfulness techniques.
**Domain**	
**Support via websites and mobile phones**	“What are your thoughts about using mobile phones and online programmes to deliver interventions?”<br>***prompt:*** *“What might be the benefits for people receiving support through a website or mobile phone?”*<br>***prompt:*** *“What concerns would you have about people*

	receiving support through a website or mobile phone?"
**Overcoming access issues:**	"Not everyone is able to get access to the support that they need and there may be waiting lists to receive support. How do you think websites and mobile phone applications could overcome problems with access to help?"
**Privacy issues:**	"Do you have any concerns about the privacy or safety of support offered through mobile phone applications or the internet?"<br>***if yes* – *prompt:*** *"can you tell me a bit more about these concerns?"*<br>***if no* – *prompt:*** *"can you tell me a bit more about why you do not have any concerns about this?"*
**Symptom monitoring:**	"There are now smartphone applications where clients can receive alerts or prompts to complete questions on the application about their thoughts and feelings a few times day; for example mood, anxiety and hallucinations, and they can then be sent graphs weekly, monthly and so on to show how they're feeling over time. What are your thoughts about this?"<br>***Prompt:*** *"What do you think might be the benefits of this type of application?"*<br>***Prompt:*** *"What concerns would you have about this type of application?"*<br>"There has also been the suggestion that the information a person gives on this sort of smartphone application could then be sent to their healthcare professionals for example their psychiatrist or care coordinator for them to have to look over. What are your thoughts about this?"<br>***Prompt:*** *"What do you think might be the benefits for you having this information?"*<br>***Prompt:*** *"What concerns would you have about you getting this information?"*

	Probe: *"Would you prefer to automatically receive this information or for clients to take the information on the smartphone directly to you themselves? Why?"*
**Healthcare professionals on social media:**	"Some people have suggested that social media accounts contain a lot of information about a person's daily life and could be used by a person's psychiatrist or care coordinator to see how their doing. What are your thoughts about healthcare professionals looking at clients social media accounts?"<br>***Prompt:*** *"What might be the benefits of you accessing this information?"*<br>***Prompt:*** *"What concerns would you have about accessing this information?"*
**Researchers on social media:**	"What some researchers are now doing is automatically collecting mental health data for example tweets from people's social media accounts for research if the account is not set to private. What are your thoughts about researchers using data from peoples social media accounts for research"?<br>***Probe:*** *"Do you have any concerns that consent is not provided?"*
**Future ideas for technology-delivered interventions:**	"Can you think of anything you would like to see in future therapies offered online or through mobile phones?"<br>***If struggling:*** *"Imagine you were creating a mobile phone application or website for your clients what sort of things would you like to see included that might be helpful?"*

Interview closedown

"Is there anything else that you would like to tell me that we haven't discussed, but you think might be relevant when thinking about mental health and the internet and mobile phones?"

“How have you found this focus group today?”
“So I’ll also be interviewing other people about their experiences and thoughts about technology and mental health. How do you think this focus group could be improved for future participants?”
“Ok I’ll now switch of the audio recorder.”

End of interview

Thank the participants for taking part.<br>Explain what will happen with the information provided.<br>Ask whether it would be ok to contact the participant in a few months to double check they agree with the interpretation of their information.<br>Ask the participant whether they would like to receive a summary of the results.<br>Ask the participant whether they have any questions.
